# CCR5 drives NK cell–associated airway damage in pulmonary ischemia-reperfusion injury

**DOI:** 10.1172/jci.insight.173716

**Published:** 2023-11-08

**Authors:** Jesse Santos, Ping Wang, Avishai Shemesh, Fengchun Liu, Tasha Tsao, Oscar A. Aguilar, Simon J. Cleary, Jonathan P. Singer, Ying Gao, Steven R. Hays, Jeffrey A. Golden, Lorriana Leard, Mary Ellen Kleinhenz, Nicholas A. Kolaitis, Rupal Shah, Aida Venado, Jasleen Kukreja, S. Sam Weigt, John A. Belperio, Lewis L. Lanier, Mark R. Looney, John R. Greenland, Daniel R. Calabrese

**Affiliations:** 1 Department of Medicine, UCSF, San Francisco, California, USA.; 2 Department of Surgery, UCSF - East Bay, Oakland, California, USA.; 3 Parker Institute for Cancer Immunotherapy, San Francisco, California, USA.; 4 Department of Microbiology and Immunology, and; 5 Department of Surgery, UCSF, San Francisco, California, USA.; 6 Department of Medicine, UCLA, Los Angeles, California, USA.; 7 Medical Service, Veterans Affairs Health Care System, San Francisco, California, USA.

**Keywords:** Pulmonology, Transplantation, Chemokines, Innate immunity, NK cells

## Abstract

Primary graft dysfunction (PGD) limits clinical benefit after lung transplantation, a life-prolonging therapy for patients with end-stage disease. PGD is the clinical syndrome resulting from pulmonary ischemia-reperfusion injury (IRI), driven by innate immune inflammation. We recently demonstrated a key role for NK cells in the airways of mouse models and human tissue samples of IRI. Here, we used 2 mouse models paired with human lung transplant samples to investigate the mechanisms whereby NK cells migrate to the airways to mediate lung injury. We demonstrate that chemokine receptor ligand transcripts and proteins are increased in mouse and human disease. CCR5 ligand transcripts were correlated with NK cell gene signatures independently of NK cell CCR5 ligand secretion. NK cells expressing CCR5 were increased in the lung and airways during IRI and had increased markers of tissue residency and maturation. Allosteric CCR5 drug blockade reduced the migration of NK cells to the site of injury. CCR5 blockade also blunted quantitative measures of experimental IRI. Additionally, in human lung transplant bronchoalveolar lavage samples, we found that CCR5 ligand was associated with increased patient morbidity and that the CCR5 receptor was increased in expression on human NK cells following PGD. These data support a potential mechanism for NK cell migration during lung injury and identify a plausible preventative treatment for PGD.

## Graphical abstract

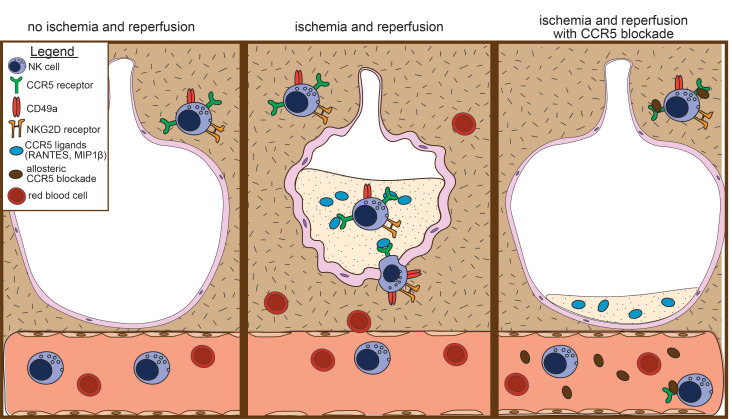

## Introduction

Lung transplantation improves quality and duration of life for many patients suffering from advanced lung disease (1). However, compared with other solid organ transplant recipients, lung transplant patient survival is limited (2). Pulmonary primary graft dysfunction (PGD) is the clinical manifestation of severe ischemia-reperfusion injury (IRI) after lung transplantation (3). PGD occurs in up to one-third of all recipients within the first 3 days after transplantation and is responsible for 50% of the first-year mortality after lung transplantation (4). Additionally, PGD is associated with lower baseline lung function and attenuated quality of life (5, 6). PGD shares basic definitions and pathophysiology with acute respiratory distress syndrome (ARDS) (7). Clinically, it is diagnosed by the presence of bilateral pulmonary opacities and ratio of arterial oxygen pressure to inspired oxygen content (PaO_2_/FiO_2_) (8–10). PGD has no effective medical therapies beyond supportive care (11, 12). Thus, identifying targetable mechanisms of PGD pathogenesis is a major imperative for improving short- and long-term lung transplantation success.

The pathophysiology of PGD is characterized by diffuse alveolar damage and follows a cascade of injury described by epithelial and endothelial injury (13), release of inflammatory mediators, and alveolar-capillary barrier disruption (14, 15). Innate immune cells, including neutrophils and macrophages, are the primary mediators of the early injury of IRI (13). We previously demonstrated a key role for natural killer (NK) cells in the development of pulmonary PGD (16). In this context, recipient NK cells traffic from the peripheral circulation to the airway lumen (16). Multiple receptors have been implicated in NK cell trafficking to sites of injury during infection, including S1P5, CCR2, CCR5, and CXCR3 (17–22). At the same time, observational studies of human lung transplant PGD patients have identified chemokines as markers of injury severity. Specifically, ligands for CCR5, including monocyte chemoattractant protein 1 (MCP1/CCL2), monocyte inflammatory protein 1a (MIP-1α/CCL3), MIP-1β/CCL4, and receptor for advanced glycosylation end products (RANTES/CCL5), have been shown in plasma or bronchoalveolar lavage (BAL) to be increased during PGD (17, 21, 23). However, it is not known how NK cells traffic to the airways during IRI and to what extent this airway localization of NK cell responses contributes to IRI pathology. Here, we tested the hypothesis that NK cell–mediated airway inflammation depends on chemotaxis though CCR5.

## Results

### Chemokine ligand transcripts are increased in the orthotopic lung transplant with prolonged cold ischemia model.

**Figure 1:**
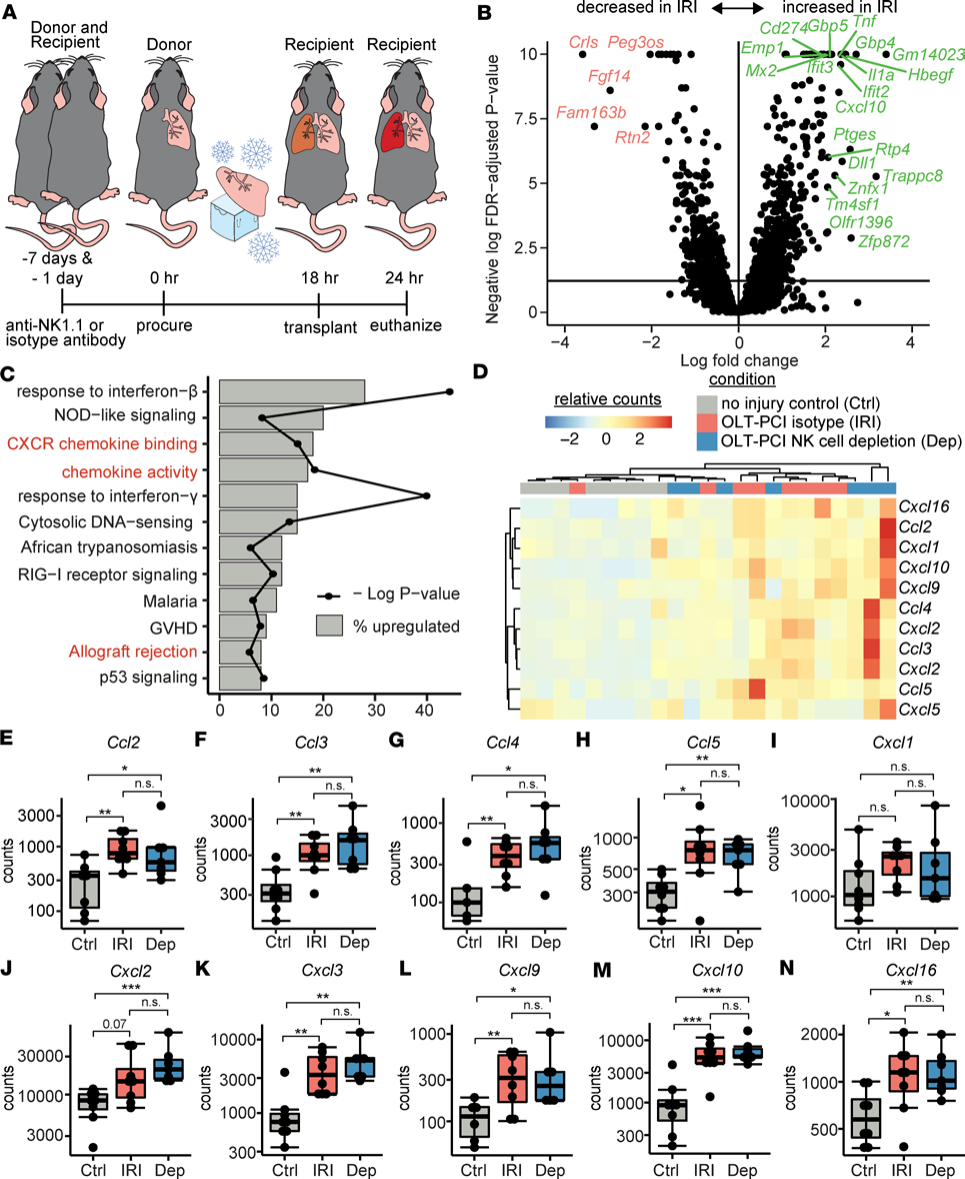
RNA sequencing following mouse orthotopic lung transplantation with prolonged cold ischemia (OLT-PCI) identifies NK cell–independent upregulation of chemokine ligands. (**A**) OLT-PCI was followed by RNA sequencing of right (Ctrl) and left (IRI) lungs from mice treated with a control antibody (*n* = 8) and left lungs (Dep) from mice treated with an NK cell–depleting antibody (*n* = 7). Data were collected 6 hours after transplantation. (**B**) Volcano plot highlighting the top 25 differentially expressed genes by log(fold change). (**C**) Differential gene transcription analyses showing KEGG and Gene Ontology pathways were enriched for those containing chemokine-associated transcripts (red). (**D**) Heatmap and hierarchical clustering of the top 11 chemokine ligand transcripts. Differences across the 3 conditions are shown for transcript counts of the following genes: (**E**) *Ccl2*, (**F**) *Ccl3*, (**G**) *Ccl4*, (**H**) *Ccl5*, (**I**) *Cxcl1*, (**J**) *Cxcl2*, (**K**) *Cxcl3*, (**L**) *Cxcl9*, (**M**) *Cxcl10*, and (**N**) *Cxcl16*. Box-and-whisker plots display individual data points bound by boxes at 25th and 75th percentiles and medians depicted with bisecting lines. Differences were assessed using the Mann-Whitney *U* test. *P < 0.05; **P < 0.01; ***P < 0.001.

We sought to perform a screening analysis for factors associated with IRI that correlate with NK cell activity independent from their function. We used a mouse model of left orthotopic lung transplantation with prolonged cold ischemia (OLT-PCI, Figure 1A), which closely replicates the conditions of human lung transplantation and would provide increased power for discovery analyses (24). Left lungs (injury) and right lungs (control) were collected from OLT-PCI mice that were treated with an isotype-matched control antibody (*n* = 8), and left lungs were also collected from OLT-PCI mice that received an anti-NK1.1 antibody (*n* = 7) for NK cell depletion. We performed RNA sequencing and analyzed differential gene expression across these 3 groups. There were 288 differentially expressed transcripts in the injured lungs compared with uninjured lungs (all false discovery rate adjusted for *P* < 0.05). Figure 1B shows the top 25 genes based on log(fold change), and Supplemental Table 1 (supplemental material available online with this article; https://doi.org/10.1172/jci.insight.173716DS1) lists the top 50 differentially expressed genes. Figure 1C illustrates the pathways with increased gene transcription in the injured lungs relative to control lungs. As reported previously, genes in type I interferon and p53 pathways were increased (25, 26). Notably, several pathways containing chemokine receptors and ligands were active during IRI. Figure 1D shows a heatmap of the top chemokine ligand transcripts across conditions, demonstrating hierarchical clustering differences between injured lungs and uninjured lungs. Individual transcript counts are displayed in Figure 1, E–N. While NK cells can secrete some chemokines, we noted that almost all chemokines were increased within the NK cell–depleted group, demonstrating that these are transcribed independently from NK cell effector function.

### Mouse and human metagenes are correlated with CCR5 receptor ligand transcripts.

**Figure 2:**
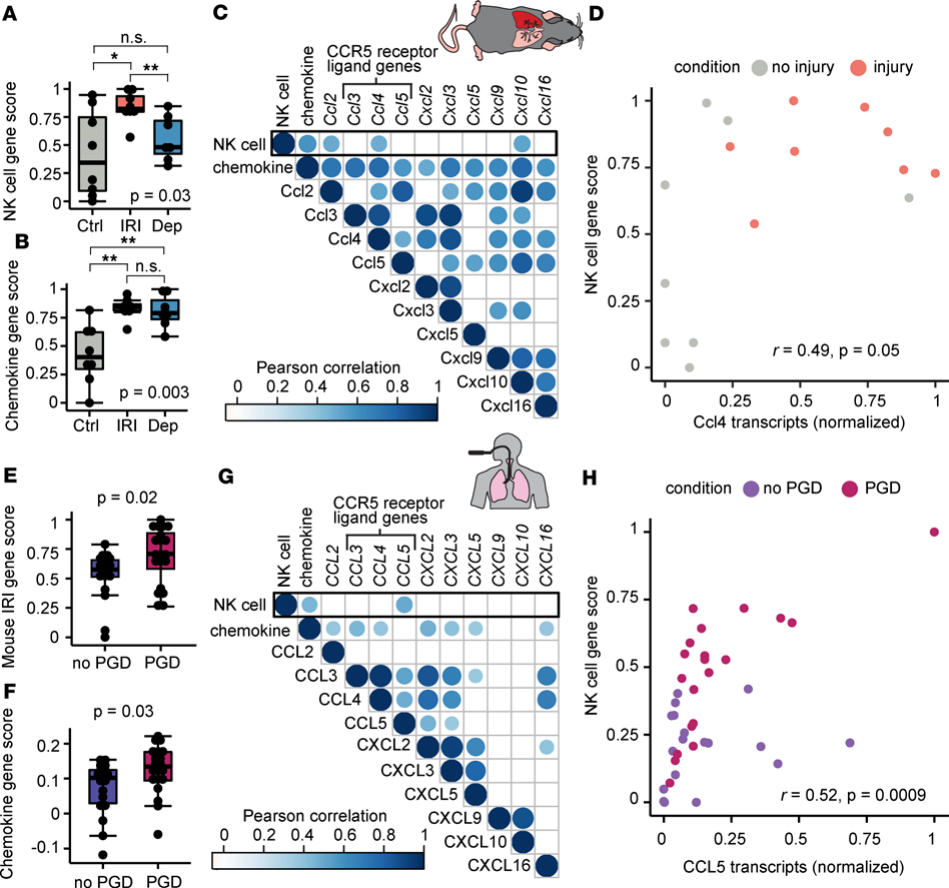
Mouse and human lung NK cell metagenes are correlated with CCR5 receptor ligand transcripts during IRI. Chemokine and NK cell metagene scores were derived from OLT-PCI RNA sequencing data shown in Figure 1. Mouse data were collected 6 hours after transplantation. (**A**) NK cell metagene score compared between OLT-PCI left lung (IRI), contralateral control (Ctrl), and OLT-PCI with NK cell depletion (Dep). (**B**) Chemokine metagene score across these 3 conditions. (**C**) Correlation matrix relating this NK cell metagene with the chemokine metagene and subcomponent chemokines, showing greatest correlation with *Ccl2*, *Ccl4*, and *Cxcl10*. (**D**) Correlation dot plot showing that NK cell gene score and *Ccl2* distinguish control from injured lungs. (**E**) Mouse IRI gene score is increased in human BAL samples with PGD. RNA sequencing was performed on human bronchoalveolar lavage collected on postoperative day 1. (**F**) Human chemokine gene score derived from most differentially expressed mouse chemokine ligands is increased in human PGD. (**G**) Human correlation matrix between NK cell gene transcripts and individual chemokines, showing greatest correlation with *CCL5*. (**H**) Correlation dot plot showing NK cell gene score and *CCL5* transcripts colored by PGD status. Box-and-whisker plots display individual data points bound by boxes at 25th and 75th percentiles and medians depicted with bisecting lines. Differences between 3 groups were assessed using the Kruskal-Wallis test. Post hoc testing and comparisons between 2 groups employed the Mann-Whitney *U* test with Benjamini-Hochberg corrections for multiple comparisons. *P* values are shown or *P < 0.05; **P < 0.01.

We were interested in screening for which chemokine transcripts were correlated to NK cell genes. To test the association between chemokine expression and NK cell recruitment, we devised separate gene scores to capture NK cell– and chemokine ligand–associated transcription within the mouse lung (Supplemental Table 2), based on prior published NK cell molecular phenotyping (27) and our chemokine transcript findings, respectively. We first assessed whether the mouse NK cell gene score captured NK cells in the isograft. Indeed, Figure 2A shows that the NK cell gene score was increased in the injured lungs (*P* = 0.02) relative to uninjured lungs and decreased in the NK cell depletion–injured lungs relative to injured lungs (*P* = 0.04). To verify that chemokine ligand mRNA transcription occurred independently from NK cells, we assessed the chemokine gene score across the 3 conditions. Indeed, injured lungs from both control antibody–treated (*P* = 0.002) mice and NK cell–depleted (*P* = 0.002) mice had increased chemokine gene scores (Figure 2B), suggesting an independent process. We assessed the correlation between individual chemokine ligand transcripts and the NK cell gene score. Figure 2C shows a correlation matrix where we found moderate correlation between NK cell gene score and *Ccl2* (Pearson’s *r* = 0.5, *P* = 0.05), *Ccl4* (*r* = 0.49, *P* = 0.05), and *Cxcl10* (*r* = 0.53, *P* = 0.04). Figure 2D shows a correlation plot illustrating that *Ccl4* mRNA, one of the ligands for the CCR5 receptor, and NK cell gene score distinguish the control antibody–treated OLT-PCI lungs from the control undamaged lungs.

We tested whether a similar approach in humans would identify potential mediators of NK cell trafficking during PGD. We reanalyzed our previously published RNA-sequencing data from BAL cells collected on the first postoperative day in lung transplant recipients with severe PGD (*n* = 19) and without PGD (*n* = 19) (28), using 3 gene scores. First, we created a human gene score from the top differentially expressed genes in our mouse model (Supplemental Table 1) and found that the mouse IRI genes were similarly increased in human PGD lungs (Figure 2E, *P* = 0.02). Similarly, a human NK cell gene score containing the mouse chemokine ligands was also increased in human PGD lungs (Figure 2F, *P* = 0.03). We applied a similar approach to our mouse correlation analysis and plotted a matrix of human chemokine gene transcripts against our previously published NK cell gene score (Figure 2G) (28). We found that expression of human BAL NK cell genes during PGD were correlated with *CCL5* transcripts, another chemokine that binds to the CCR5 receptor (Figure 2H; *r* = 0.52, *P* = 0.0009). Together, these data demonstrate that human and mouse IRI have similar pathways of lung injury, marked by chemokine transcription. These analyses determined that NK cell lung trafficking may be dependent on CCR5 receptor-ligand interactions.

### Mouse and human CCR5 ligand protein is increased in BAL during IRI and predicts clinical outcomes.

**Figure 3:**
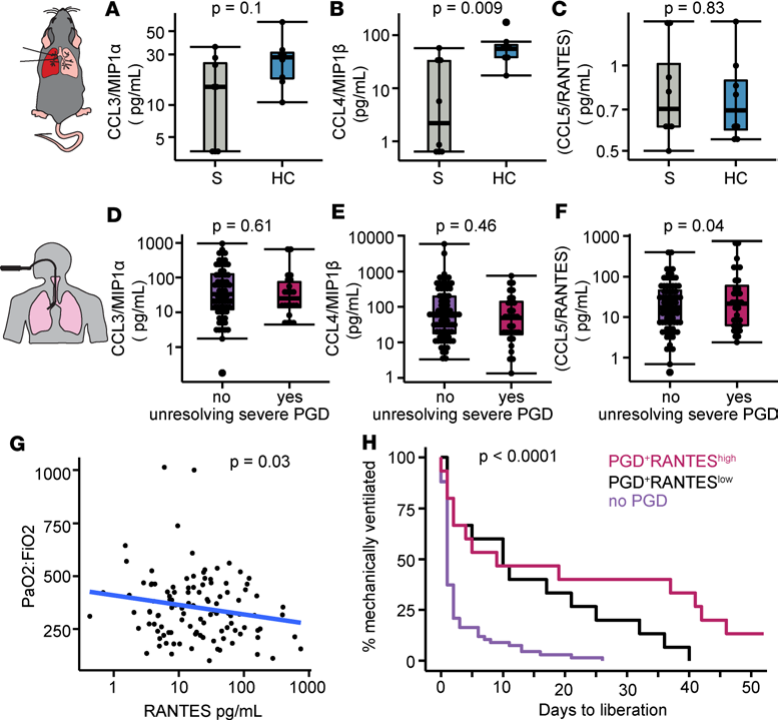
Mouse and human CCR5 ligand proteins are increased in BAL during mouse and human IRI and predict clinical outcomes. We collected left lung BAL 4 hours after hilar suture removal in the mice subjected to HC (*n* = 8) compared to sham (S, *n* = 8) procedures and measured BAL concentrations for CCR5 ligand proteins (**A**) CCL3/MIP1α, (**B**) CCL4/MIP1β, and (**C**) CCL5/RANTES. In humans, we collected BAL on the first postoperative day following lung transplantation in 34 recipients with severe PGD and 77 recipients without PGD (grade 0 or 1). We measured CCR5 ligand proteins (**D**) CCL3/MIP1α, (**E**) CCL4/MIP1β, and (**F**) CCL5/RANTES. (**G**) BAL CCL5/RANTES is inversely correlated with PaO_2_/FiO_2_ ratio on day 1. (**H**) Kaplan-Meier plot of mechanical ventilation time stratified by BAL CCL5/RANTES concentration and PGD. Summary data are displayed with box-and-whisker plots illustrating individual data points, bound by boxes at 25th and 75th percentiles, and with medians depicted with bisecting lines. Individual *P* values as assessed with Mann-Whitney *U* test (**A**–**C**), generalized linear models adjusted for recipient baseline characteristics (**D**–**G**), or log-rank test for Kaplan-Meier plot (**H**).

Given our transcriptional observations, we hypothesized that CCR5 receptor ligand proteins would be increased in BAL during IRI. In the higher-throughput hilar clamp (HC) versus sham model of pulmonary IRI, we performed left lung saline lavage and measured CCR5 receptor ligand proteins (CCL3/MIP1α, CCL4/MIP1β, and CCL5/RANTES) in supernatant. Compared with MIP1α concentrations in sham control BAL (14.9 pg/mL, IQR 3.7–24.9 pg/mL), we found no difference in HC BAL (28.3 pg/mL, IQR 17.9–31.3 pg/mL, *P* = 0.1; Figure 3A). MIP1β was increased in HC BAL (55.1 pg/mL, IQR 38.1–65.2 pg/mL, *P* = 0.008; Figure 3B) compared with sham control BAL (3.3 pg/mL, IQR 0.6–32.7 pg/mL). RANTES was not increased in HC BAL (0.7 pg/mL, IQR 0.6–0.9 pg/mL, *P* = 0.83; Figure 3C) compared with sham BAL (0.7 pg/mL, IQR 0.6–1 pg/mL).

We further hypothesized that CCR5 receptor ligand proteins would be increased in human BAL during severe PGD. Table 1 shows the baseline characteristics for the lung transplant recipients with BAL collected on the first posttransplant day available for analysis. We found that 34 participants had severe unresolving PGD (grade 2 or 3 throughout the first 72 hours). Notably, participants with severe PGD were more likely to need a transplant for interstitial lung disease (ILD) (*P* = 0.04). We found no differences in BAL concentrations of MIP1α (Figure 3D, *P* = 0.61) or MIP1β (Figure 3E, *P* = 0.46) among recipients with severe PGD compared to recipients without PGD. However, we found that CCL5/RANTES was increased in BAL from recipients with severe PGD (21.6 pg/mL, IQR 6.3–60 pg/mL, *P* = 0.04; Figure 3F) compared with BAL from recipients without PGD (16.8 pg/mL, IQR 7.1–45.8 pg/mL). On the day of BAL collection, we also found that higher arterial oxygen (PaO_2_) relative to inspired oxygen content (FiO_2_) was associated with lower RANTES concentrations (Figure 3G, *P* = 0.03), suggesting CCL5/RANTES (gene *CCL5*) is associated with ventilation. Finally, we hypothesized that CCL5/RANTES would be associated with risk for mechanical ventilation. We stratified the population of recipients with severe PGD by median BAL CCL5/RANTES concentrations. In the first 60 days after lung transplantation, recipients with severe PGD and lower than median CCL5/RANTES had 2.6 times the increased risk (95% CI 1.4–5, *P* = 0.004) for mechanical ventilation compared with recipients without PGD (Figure 3H). However, the recipients with severe PGD and higher median CCL5/RANTES concentration had nearly 5 times increased risk for invasive mechanical ventilation (HR 4.8, 95% CI 2–10.3, *P* = 0.0003). Together, these data confirm the transcriptional chemokine findings and suggest that CCR5 receptor ligands may be implicated in IRI pathology.

### CCR1 and CCR5 receptors are increased on lung NK cells following mouse IRI.

We hypothesized that pulmonary NK cells would have increased expression of chemokine receptors following IRI. We performed HC (*n* = 5) and sham (*n* = 5) procedures on mice and assessed immune cell phenotypes in the left lung, thoracic lymph node, spleen, and blood with spectral flow cytometry. Innate and adaptive immune cells were quantified broadly (Supplemental Figure 2), but we focused on NK cell phenotypes. There were no differences in NK cells frequencies in blood or spleen during HC, but we found increased frequencies of NK cells in thoracic lymph nodes and lung tissue compared with sham controls (Supplemental Figure 3).

**Figure 4:**
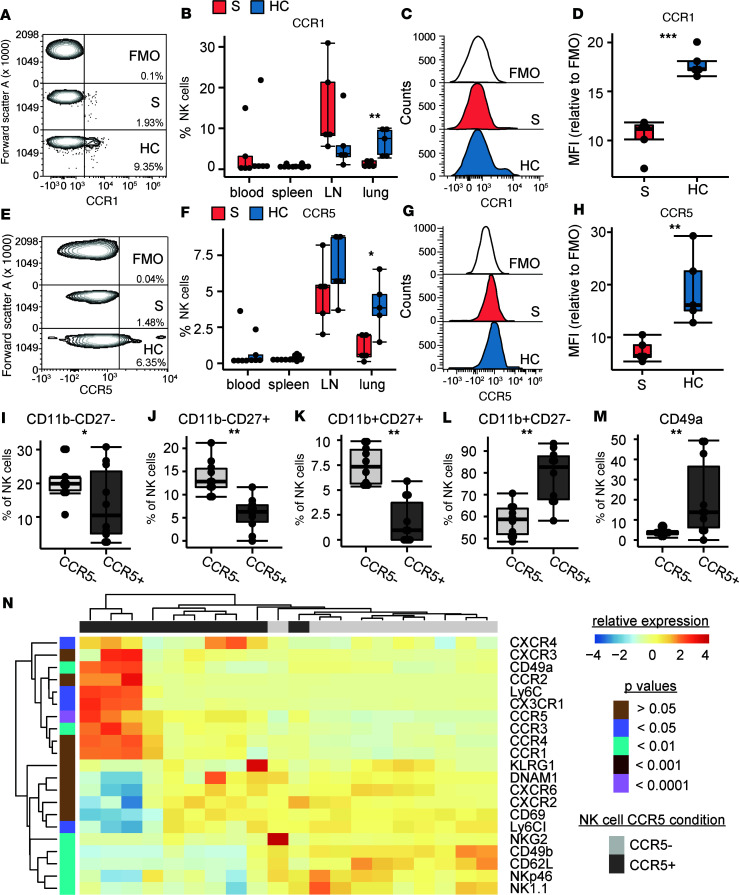
CCR5^+^ NK cells are increased during mouse HC and express markers of maturity and tissue residence. We performed HC (*n* = 5) and sham (S, *n* = 5) procedures and quantified NK cells and their phenotypes via spectral flow cytometry across blood, spleen, thoracic lymph node (LN), and lung tissues collected 4 hours after reperfusion. (**A**) Contour plot of CCR1 on NK (CD45^+^CD3^–^F480^–^CD19^–^NK1.1^+^NKp46^+^) cells in the lung. (**B**) CCR1 frequency of total NK cells during HC or S procedures. (**C**) Histograms of CCR1 on NK cells with fluorescence minus one (FMO) control, HC, and S. (**D**) CCR1 on NK cells by median fluorescence intensity (MFI). (**E**) Contour plot of CCR5 on NK cells in the lung. (**F**) CCR5 frequency of total NK cells during HC or S procedures. (**G**) Histograms of CCR5 on NK cells with FMO control, HC, and S. (**H**) CCR5 on NK cells by MFI. (**I**–**L**) We quantified maturation states of CCR5^+^ and CCR5^–^ NK cells. (**M**) Frequencies of CD49a on CCR5^+^ and CCR5^–^ NK cells. (**N**) Heatmap of MFIs of additional markers of NK cell activation. Summary data are displayed with box-and-whisker plots illustrating individual data points, bound by boxes at 25th and 75th percentiles, and with medians depicted with bisecting lines. Differences were assessed using the Mann-Whitney *U* test with Benjamini-Hochberg corrections for multiple comparisons. *P < 0.05; **P < 0.01; ***P < 0.001.

We found increased frequencies of CCR1^+^ NK cells in the lung during HC (7.3%, IQR 3.5%–9.3%) compared with sham (0.9%, IQR 0.7%–1.9%, *P* = 0.008; Figure 4, A and B). We also found increased surface CCR1 by median fluorescence intensity (MFI) on NK cells from HC lungs relative to sham lungs (*P* = 0.003; Figure 4, C and D). Similarly, we found increased frequencies of CCR5^+^ NK cells in the lung during HC (4%, IQR 3.4%–4.8%) compared with sham (0.7%, IQR 0.5%–1.9%, *P* = 0.03; Figure 4, E and F). We also found increased surface CCR5 by MFI on NK cells from HC lungs relative to sham-treated lungs (*P* = 0.008; Figure 4, G and H). Supplemental Figure 4 shows frequencies of other chemokine receptors on mouse NK cells by tissue type. In unsupervised clustering analyses, we observed that chemokine receptors were often coexpressed within activated NK cell phenotypes (Supplemental Figure 5).

We further examined the activating, inhibiting, and tissue-resident markers on chemokine-expressing NK cells to glean insights into their potential function. NK cell CCR1 analyses are shown in Supplemental Figure 6. However, we focused our studies on CCR5, given the findings of increased CCR5 ligand transcripts in mouse and human IRI BAL. NK cells change their surface markers as they mature, with the most immature NK cells being CD27^–^CD11b^–^. NK cells will first gain CD27 expression (CD27^+^CD11b^–^) and then gain CD11b expression (CD27^+^CD11b^+^), with the most mature NK cells described as CD27–CD11b^+^ as they lose the previously gained CD27 surface marker (16). We noted that CCR5^+^ NK cells had markedly mature phenotypes (Figure 4, I–L). In addition, CCR5^+^ NK cells had increased expression of CD49a (13.81%, IQR 6.2%–36.5%), a marker of tissue residency, compared with CCR5^–^ NK cells (3.2%, IQR 2.8%–4.2%, *P* = 0.0003; Figure 4M). The heatmap in Figure 4N depicts our findings that CCR5^+^ NK cells had increased expression of chemokine receptors (CXCR4, CX3CR1, CCR3, and CCR4), and activation markers (Ly6C, NKG2A, CD49b, NKp46, and NK1.1). These data suggest that CCR5 may be a marker for NK cell trafficking to the lung during sterile lung injury.

### CCR5 blockade reduces NK cell trafficking to the airways during mouse pulmonary IRI.

**Figure 5:**
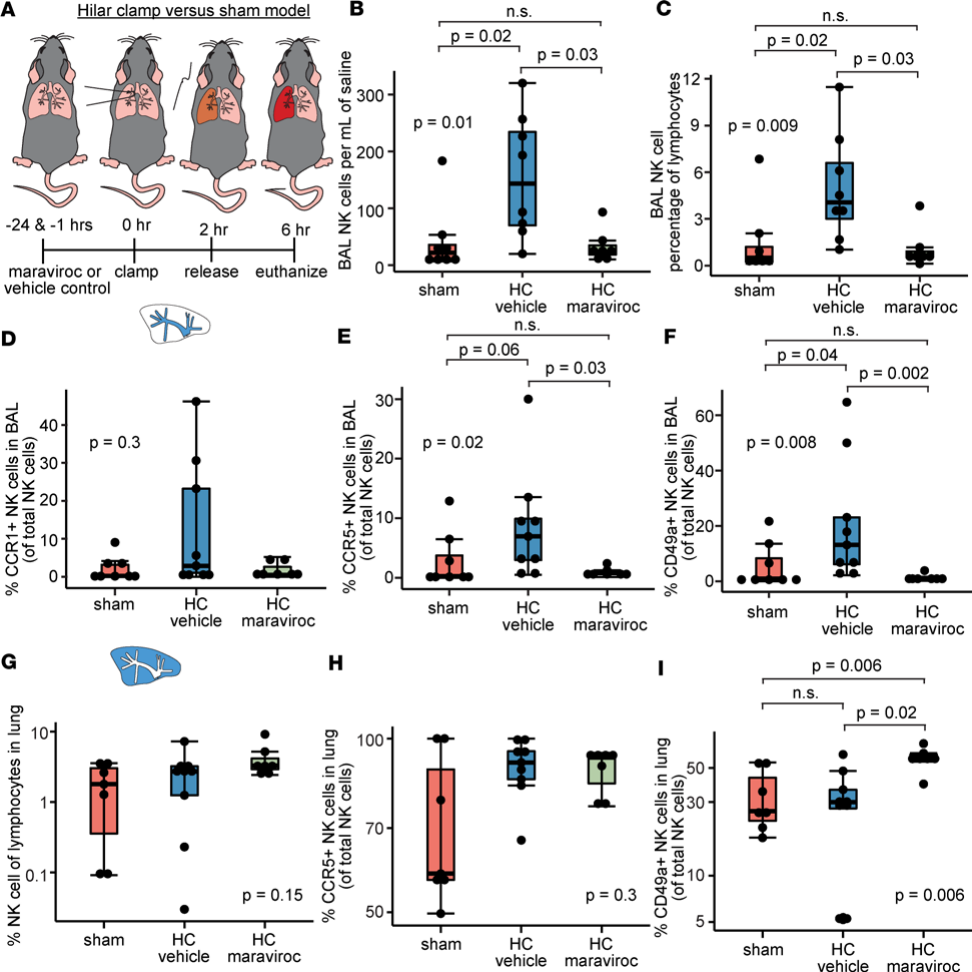
CCR5 blockade reduces mouse NK cell airway inflammation. (**A**) Schematic of maraviroc (allosteric CCR5 antagonist) administration 24 hours and 1 hour before left lung HC and reperfusion (*n* = 7) compared to vehicle control and HC (vehicle, *n* = 8) and sham procedures (*n* = 8). Samples were collected 4 hours after hilar suture removal (reperfusion). (**B**) Absolute number of NK cells within left lung BAL samples per mL. (**C**) NK cells as a percentage of total BAL lymphocytes. (**D**) Percentage of NK cells with CCR1 in the BAL. (**E**) Percentage of NK cells expressing CCR5 in the BAL. (**F**) Percentage of NK cells expressing CD49a in the BAL. (**G**) NK cells as a percentage of total lymphocytes in the lung. (**H**) Percentage of CCR5^+^ NK cells in the lung tissue. (**I**) Percentage of CD49a^+^ NK cells in the lung tissue. Summary data are displayed with box-and-whisker plots illustrating individual data points, bound by boxes at 25th and 75th percentiles, and with medians depicted with bisecting lines. Differences were assessed using the Kruskal-Wallis test. Post hoc testing between groups employed the Mann-Whitney *U* test with Benjamini-Hochberg corrections for multiple comparisons. *P* values are directly shown.

We hypothesized that blockade of the CCR5 receptor would reduce NK cell trafficking to the lungs and airways during IRI. Maraviroc is an allosteric inhibitor of CCR5 that is gaining interest in the transplant community for its immunomodulatory properties and the potential to prevent graft rejection (29–31). Most studies focused on the ability of CCR5 to modulate T cell activity; our goal was to investigate NK cell migration (32, 33). As such, we treated mice with maraviroc or vehicle control preceding HC lung injury (Figure 5A). We measured NK cells within the airways via BAL and in the remaining lung tissue compartment and identified the CCR1^+^, CCR5^+^, and CD49a^+^ subsets (Supplemental Figure 7). We found that absolute quantities of NK cells were increased during HC relative to sham lungs, but that maraviroc treatment blunted the numbers of NK cells in the BAL following IRI (Figure 5B). We discovered a similar phenomenon when NK cells were quantified as a frequency of total NK cells in the lung (Figure 5C). This suggested that maraviroc reduced NK cell migration from the lung tissue into the airways during IRI. Notably, we found no difference in CCR1^+^ NK cells in the BAL across the 3 conditions (Figure 5D). However, we found reduced CCR5^+^ NK cells in the BAL in maraviroc-treated mice relative to vehicle control mice (Figure 5E). Maraviroc also reduced the migration of tissue-resident (CD49a^+^) NK cells into the BAL (Figure 5F). Interestingly, we found little effect of maraviroc on the NK cells within the lung tissue compartment as a frequency of total lymphocytes (Figure 5G) or when we quantified the proportion of NK cells bearing CCR5 (Figure 5H) or CD49a (Figure 5I). We also assessed differences in B cells, T cells, myeloid cells, and NKp46^+^ T cells across these 3 conditions (Supplemental Figure 8), where we found no differences in quantities of these airway cells between the maraviroc-treated mice compared to vehicle control mice.

To assess whether an allogeneic transplant would have a differential impact on NK cells in the lung relative to a syngeneic transplant, we assessed donor and recipient origin via CD45 allotype (Supplemental Figure 9, A and B). We found that more NK cells were in lung allografts compared with isografts and that this effect was amplified when mice were preconditioned with IL-15, an NK cell stimulatory factor (Supplemental Figure 9C). Notably, there were fewer donor NK cells in allografts (Supplemental Figure 9D), which largely reflected an influx of recipient cells (Supplemental Figure 9E). Together, these data suggest that NK cells migrate to the airways, potentially from lung tissue reservoirs, in a CCR5-dependent manner and that these effects may be amplified in allografts relative to isografts.

### CCR5 blockade reduces lung damage during mouse pulmonary IRI.

**Figure 6:**
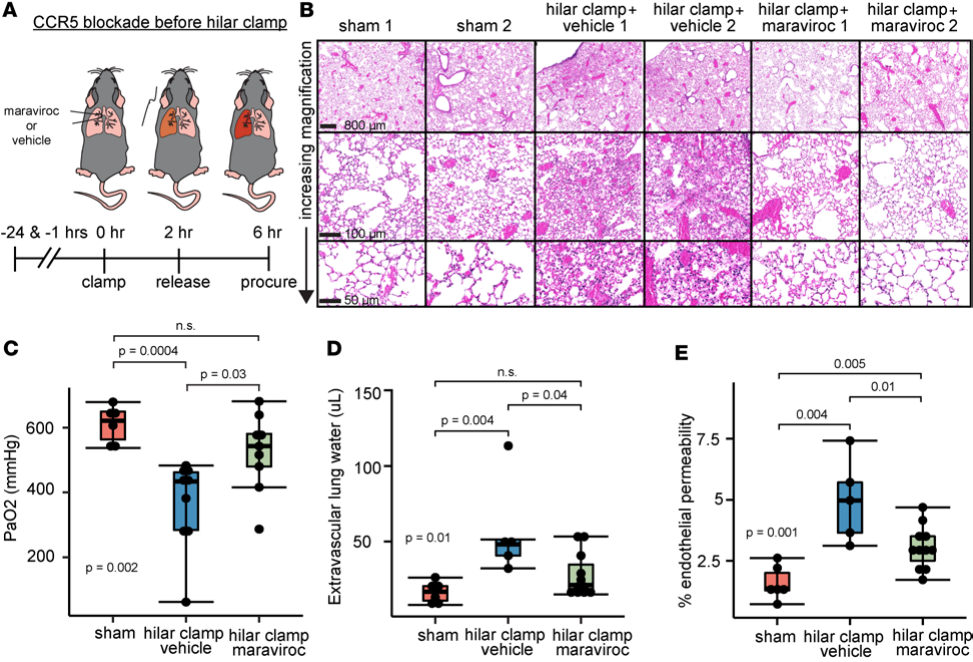
CCR5 blockade reduces lung damage during mouse pulmonary IRI. (**A**) Schematic of maraviroc (allosteric CCR5 antagonist) administration 24 hours and 1 hour before left lung HC and reperfusion compared to vehicle control with HC and sham procedures. Injury was assessed 4 hours after left hilar suture removal. (**B**) Representative H&E staining. Scale bars: 800 μm (top row), 100 μm (middle), and 50 μm (bottom). We also performed quantitative measures of lung injury: (**C**) partial pressure of oxygen in mmHg (PaO_2_), (**D**) extravascular lung water (μL), and (**E**) percentage of endothelial permeability. Experiments studied at least *n* = 6 animals per condition. Summary data are displayed with box-and-whisker plots illustrating individual data points, bound by boxes at 25th and 75th percentiles, and with medians depicted with bisecting lines. Differences were assessed using the Kruskal-Wallis test. Post hoc testing between groups employed the Mann-Whitney *U* test with Benjamini-Hochberg corrections for multiple comparisons. *P* values are directly shown.

We previously observed that lung injury during IRI was dependent on NK cell recruitment and activation. Here, we hypothesized that blockade of CCR5 would reduce lung damage during IRI. To address this hypothesis, we again treated mice with maraviroc or vehicle control preceding HC lung injury and compared these findings against sham-injured animals (Figure 6A). We found that maraviroc treatment decreased IRI pathology compared with vehicle-treated HC lungs, noted by reduced alveolar interstitial thickening, hemorrhage, neutrophil infiltration, and hyaline membrane formation (Figure 6B). Quantitative measures of lung injury supported these representative pathologic findings. We found improved PaO_2_ (Figure 6C) and less extravascular lung water (Figure 6D) in maraviroc-treated animals compared with injured animals, and both of these measures were not different compared to sham lungs. For endothelial permeability (Figure 6E), we found that maraviroc treatment reduced measures of injury compared with vehicle control HC animals. However, the maraviroc-treated animals had increased injury relative to sham by this measure. We conclude that maraviroc blunts key metrics of acute lung injury induced by ischemia and reperfusion, likely resulting from a reduction in NK cell airway trafficking.

### CCR5 NK cells are increased and activated in lung transplant recipient BAL during human PGD.

**Figure 7:**
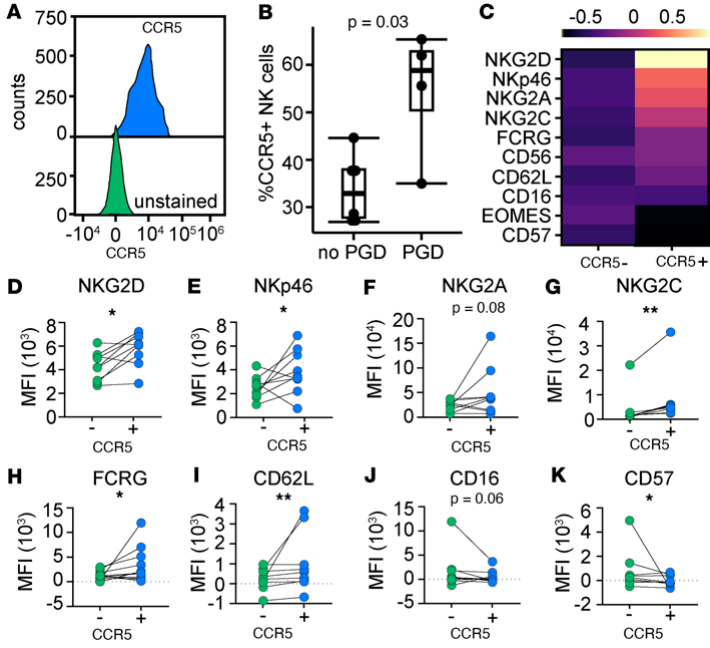
CCR5^+^ NK cells are increased in human BAL during PGD. Human BAL samples were prospectively collected from lung transplant patients with severe PGD (*n* = 4) and those without PGD (*n* = 6) at 2 weeks after transplant. (**A**) Histogram demonstrating CCR5 staining versus unstained control on NK cells from BAL samples. (**B**) Percentage of CCR5^+^ NK cells in BAL. (**C**) Heatmap of surface marker MFI differences between CCR5^+^ and CCR5^–^ NK cells with individual plots of surface markers on NK cells stratified by CCR5 shown for (**D**) NKG2D, (**E**) NKp46, (**F**) NKG2A, (**G**) NKG2C, (**H**) FCER1G (FCRG), (**I**) CD62L, (**J**) CD16, and (**K**) CD57. Summary data are displayed with individual data points alone (**D**–**K**) or bound by boxes at 25th and 75th percentiles, and with medians depicted with bisecting lines (**B**). Differences were assessed with generalized linear models adjusted for recipient baseline characteristics. *P < 0.05, **P < 0.01.

We have previously shown that BAL NK cells are increased and activated in human BAL within the first 90 days after lung transplantation in recipients who developed severe PGD. Here, we hypothesized that BAL NK cells, collected within the first 2 weeks after transplant, would have increased CCR5. Supplemental Table 3 shows the demographics for this cohort of recipients and the gating strategy is displayed in Supplemental Figure 10. We measured CCR5 on NK cells in BAL (Figure 7A). We found that the frequency of NK cells with CCR5 was increased in BAL of recipients with severe PGD (58.8%, IQR 50.5%–62.9%, *P* = 0.03) as compared with recipients without PGD (32.9%, IQR 27.7%–37.9%; Figure 7B). We also report significant differences in expression of activating receptors on CCR5^+^ compared with CCR5^–^ NK cells (Figure 7C) (34). Notably, 2 activating receptors involved in the NK cell damage response, NKG2D (Figure 7D) and NKp46 (Figure 7E), were increased on CCR5^+^ NK cells. We also quantified NKG2A (Figure 7F), NKG2C (Figure 7G), FCER1G (Figure 7H), CD62L (Figure 7I), CD16 (Figure 7J), and CD57 (Figure 7K) (35). These data corroborate our findings in the mouse and suggest that NK cells may migrate to human airways, as determined by BAL concentration, in a CCR5-dependent manner.

## Discussion

We observed that CCR5 ligands and proteins were increased during IRI across 2 different experimental models and within a multicentric observational human cohort. NK cells bearing tissue-resident markers were abundant at the site of IRI, but their migration to the airways depended on chemotaxis through the CCR5 receptor. These CCR5^+^ NK cells also expressed high levels of NKG2D, suggesting that CCR5 blockade may target the NK cell–mediated injury linked to stress molecule recognition. These findings contribute to the hypothesis that NK cells mediate acute lung injury pathogenesis via damage to the airways and underscore the importance of the epithelial cell–innate immune cell interface in the lung. Further, these data suggest CCR5 blockade is a plausible intervention for reducing NK cell inflammation during human acute lung injury.

There is an emerging understanding that the airway epithelium plays an active role in driving pulmonary IRI pathology (3, 36). Compared with endothelial cells, lung epithelial cells have more robust expression of stress ligands for the NKG2D receptor in response to IRI in mice (16). In human PGD, airway epithelial cells were also found to be the dominant source of cellular stress markers for NKG2D-dependent NK cell activation (28). Epigenetic studies of lung transplant airways reveal that PGD is associated with accelerated aging and reprogramming toward a proinflammatory state (37). IRI induces airway epithelial cell swelling, membrane thickening, accumulation of reactive oxidation species, and necroptotic cell death (38). The finding that abrogation of lung injury occurred through blunting of NK cell migration to the airways supports the notion that IRI pathology may develop from the interaction between airway insult and NK cell activation. Such activation may help explain the link between PGD and the bronchiolar pathology associated with chronic lung allograft dysfunction (39).

Tissue-resident innate lymphoid cells (ILCs) are increasingly recognized as critical for mucosal health. In lung transplantation, donor tissue NK cells and ILC1s are altered in patients with severe PGD (40). Conversely, ILC2s and ILC3s may contribute to lung transplant tolerance through a variety of mechanisms (41, 42). Here, we found that CD49a^+^ NK cells, likely tissue-resident in origin, trafficked into the airways as they constituted a significant proportion of the sampled BAL NK cells. The tissue-resident NK cell population likely plays an important role in disease-specific innate memory (43). We further found that CCR5 blockade reduced the CD49a^+^ NK cell population within the BAL, suggesting that CCR5 may be a mechanism for NK cell homing to sites of injury and establishment of tissue residency (18). Indeed, CCL5/RANTES is secreted by airway epithelial cells during injury (44). In kidney transplant models, CCR5 has been shown to be important for NK cell function in a myeloperoxidase-dependent fashion, which may also explain some of the abrogation of lung injury we observed in maraviroc-treated mice (45). Further, as cytomegalovirus (CMV) remains a major pulmonary infection after transplantation, this work provides a potential mechanism whereby CMV-specific NK cells may migrate to areas of active replication.

These results are broadly applicable to a range of other acute lung syndromes. In support of our findings here, serial plasma samples demonstrated that CCL5/RANTES is associated with severe PGD (46). CCL5/RANTES has been associated with severity of lung damage in ARDS, a clinical syndrome of severe acute respiratory failure, and is an independent risk factor for development of acute lung injury (47). CCL5/RANTES may be pathogenic in lungs with severe COVID-19 (48, 49). Our previous finding that transcriptional programs found in ARDS airways are also present in PGD suggest a central program of injury pathogenesis between PGD and ARDS (16). Thus, CCR5 receptor-ligand interactions may be a common mechanism underlying acute lung injury independently of etiology.

Our finding that differentially expressed mouse injury transcripts are also increased in human lung transplant BAL during PGD provides important real-world validation that our model system reflects human biology. It is important to highlight that our orthotopic transplant models were syngeneic, whereas others have used major- and minor-antigen-mismatched allogeneic donors (50, 51). We interpret the transcriptional concordance between the syngeneic mouse system and human BAL samples to indicate that much of the early damage of IRI is driven by innate inflammation. Here, we found different CCR5 ligands in mouse and human. This may stem from sampling time differences within the respective species: 8 hours versus 24 hours in mice and humans, respectively. Notably, we used 2 models of IRI in this study: an OLT-PCI model to screen for candidate chemokine transcripts, and a warm IRI model to study functional differences in immune cells and between experimental interventions. While both models are endorsed via consensus opinion, there may be important differences in NK cell function and trafficking within lungs injured via prolonged cold ischemia warranting additional study (51).

Currently there are no approved or successful therapies for the targeted modulation of NK cell activity. In fact, NK cells are resistant to common immunosuppression regimens as they are less dependent on transcription for their cytotoxicity (52). Thus, the finding that CCR5 blockade blunted NK cell inflammation represents key progress in the field. Maraviroc is an FDA-approved agent targeting this CCR5 pathway that has demonstrated broad safety across a range of at-risk patient groups (53). Maraviroc is metabolized separately from common induction and maintenance immunosuppression agents used in solid organ transplantation and has not been shown to interfere with therapeutic drug levels (54). Importantly, kidney and heart allogeneic transplant models have demonstrated increased antibody-mediated rejection in the chronic absence of CCR5 signaling (45, 55, 56). While the short-term effects of CCR5 blockade on humoral rejection have not been elucidated, humoral activation will be an important safety endpoint for translation to thoracic transplantation. We found no differences in other airway cells with CCR5 blockade; however, CCR5 likely has pleiotropic effects in the lung across a wide range of target cells. While we demonstrate reduced NK cell airway trafficking during CCR5 blockade, the reduction in lung injury may also result from alternate pathways. As such, more work is needed to identify NK cell CCR5-dependent and independent effects. Finally, there are active clinical trials in kidney transplant recipients with HIV examining the utility of a maraviroc-inclusive posttransplant drug regimen. The present data support further investigation of maraviroc in the context of lung transplantation as well.

This study has some limitations. The human BAL was collected with 10–20 mL of saline, depending on final sample volume; however, it may be that participants with severe PGD had less volume instilled. As we were unable to adjust protein concentration for installation volume, our findings may be biased to the null hypothesis. While human data on BAL NK cells were consistent with our observations in mouse models, we do not have cellular samples from humans before 2 weeks after transplantation. Conversely, the mouse OLT-PCI models do not incorporate allogeneic differences observed in PGD. Differences in donor and recipient major histocompatibility complex have been shown in mouse models and human lung transplant registry data to drive tolerance through selective donor antigen-presenting cell depletion (57, 58). Thus, NK cell function in allogeneic models of cold static storage and the role of CCR5 blockade in mediating tolerance and lung injury require further investigation. Our focus was on NK cell migration to the airways, but a broader assessment of CCR5 effects may yield additional insights into IRI pathophysiology.

In summary, we describe an important mechanism whereby NK cells migrate to the airways during pulmonary IRI. Our findings in mice and humans implicate the CCR5 receptor as a rational target for further investigation as a complement to lung transplant induction immunosuppression regimens.

## Methods

### Mice.

Male mice aged 8 to 12 weeks and weighing 25–30 grams were housed in a pathogen-free barrier facility for all experiments. C57BL/6J (stock 000664), BALB/cJ (stock 000651), and C57BL/6J CD45.1 (B6.SJL-*Ptprc^a^* *Pepc^b^*/BoyJ, stock 002014) animals were purchased from The Jackson Laboratory.

### Experimental models of pulmonary IRI.

Pulmonary IRI model techniques were performed according to our previously published methods (16, 24). Mouse procedures were performed by an experienced microvascular surgeon. For the warm IRI model, mice underwent a left thoracotomy, and a suture was tied in a slipknot around the left hilum (hilar clamp, HC) or left untied in a sham surgery. Following 2 hours of ischemia, the suture was removed, and animals were euthanized after 4 hours of reperfusion. In a series of experiments, 10 mg/kg maraviroc (S2003, Selleck Chemicals) or a vehicle control (castor oil) was injected 24 hours and 1 hour prior to HC.

We also performed syngeneic OLT-PCI, as previously reported (24). C57BL/6J donor and recipient mice received intraperitoneal (i.p.) doses of 200 μg anti-NK1.1 monoclonal antibody (HB-191, clone PK136, American Type Culture Collection) in saline or control antibody (anti-rat gp42 IgG2a, clone 3G7, provided by Mary Nakamura, UCSF and San Francisco VA Medical Center) at 7 days and 24 hours preceding surgery (16, 59). Briefly, the left donor lung was inflated and infused with Perfadex (XVIVO Perfusion Inc.) and stored at 4°C for 18 hours before implantation using a plastic cuff for the anastomosis. The recipient animal was euthanized 8 hours after transplantation. The left and right lungs were collected for analysis. Dissociated native and uninjured lungs were used as internal flow cytometry controls.

In a separate series of experiments, C57BL/6J CD45.1 recipient mice were treated with 10 mg/kg IgG2a isotype control (clone C1.18.4, BioXcell, BE0085), or 0.4 mg/kg IL-15 receptor complex 1 hour before OLT with a BALB/c donor lung. IL-15 was complexed prior to injection by mixing 1.5 μg of mouse recombinant IL-15 (R&D Systems, 447ML010CF) with 10.05 μg of mouse recombinant IL-15Rα-Fc (R&D Systems, 551MR100) and incubated at 37°C for 20 minutes. Lungs were collected 3 days later for immunophenotyping.

### Acute lung injury measurements, BAL collection, and cytokine assessments.

Lung vascular permeability to ^125^I-labeled albumin (Jeanatope, Iso-Tex Diagnostics) delivered retro-orbitally, and extravascular lung water, and gamma counts of ^125^I-labeled albumin were measured as previously described (16). Arterial blood PaO_2_ was also assessed with an i-STAT 1 Handheld Analyzer and VetScan CG4 i-STAT Cartridges (Abaxis, catalog 89126). In representative conditions, mice were euthanized and lungs were inflated with 1% paraformaldehyde in PBS, stored in 70% ethanol, and embedded in paraffin before sectioning and hematoxylin and eosin (H&E) staining. Digital slide images were scanned with a Zeiss Axio ScanZ.1 microscope and visualized with ImageJ software (60). Single left lung lavage with 200 μL sterile saline was performed, cells were pelleted for 5 minutes at 300*g* at 4°C, and supernatants were cryopreserved. BAL chemokine proteins were measured with a multiplex Luminex-based assay (Eve Technologies).

### Lung digestion.

To isolate cells from harvested lungs, the left lungs were added to a solution containing 1 mL PBS with 10 μL of 10 μg/μL collagenase D (Sigma-Aldrich, 11088858001) and 10 μL of 10 μg/μL DNase I (Sigma-Aldrich, 10104159001). The lungs were mechanically dissociated with scissors and incubated in a shaking incubator for 20 minutes at 37°C. The resultant material was strained through a 40-μm nylon mesh filter and pelleted for 5 minutes at 300*g* and 4°C. The samples were resuspended in 2 mL PBS and 2 mL ACK Lysis Buffer (A10492, Gibco) for 5 minutes at room temperature, and then pelleted for 5 minutes at 300*g* and 4°C. Each sample was then washed and resuspended in flow cytometry buffer (BD, E554656).

### Mouse lung immunophenotyping.

For broad chemokine receptor investigations of NK cells*,* digested lung tissue cells were washed and incubated at 37°C with anti–mouse CD16/CD32 (BioLegend, 101320) to block nonspecific binding and stained with viability exclusion dye and anti-chemokine antibodies for 30 minutes. Cells were further stained with the remaining fluorophore-conjugated antibodies at 4°C, as detailed in Supplemental Table 4, for another 30 minutes. Cells were washed, fixed with Fluorofix (BD, 422101) and data were acquired on an Aurora spectral flow cytometer (Cytek).

Separately, we quantified differences in the BAL and lung tissue compartments. After Fc blockade, samples were stained with antibodies detailed in Supplemental Table 5. Flow cytometry data analysis was performed using FCS express version 7 (De Novo Software). The gating strategies for spectral flow cytometry manual analysis are shown in Supplemental Figure 2 and for the BAL and tissue immunophenotyping are shown in Supplemental Figure 7. Target markers were quantified as a percentage of total NK cells and by median fluorescence intensity (MFI). Dimensionality reduction was performed and t-distributed stochastic neighbor embedding (t-SNE) plots were generated for clustering analyses, as previously described (16). Donor and recipient NK cell immunophenotyping in the allogeneic OLT model was performed as previously described (16).

### Mouse lung RNA sequencing.

OLT-PCI lungs were collected, centrifuged through a QIAshredder (79656, Qiagen), and RNA was cryopreserved with QIAzol (79306, Qiagen). RNA was extracted with the miRNeasy Kit (Qiagen) and quality was confirmed via NanoDrop (Thermo Fisher Scientific) and Agilent 5200 Fragment Bioanalyzer (Agilent Technologies). Libraries were generated using NEBNext Ultra II Library Prep Kits (New England Biolabs) per manufacturer’s protocols and sequencing was performed on a NovaSeq 6000, as previously described (61). After alignment to the mouse genome, gene counts were normalized with DESeq and outliers were excluded using Tukey’s fence criteria (*k* > 3). Metagene values were calculated using the singscore R module (62).

### Human participant study design.

All human samples were collected from consenting adults. Protein and genomic data were obtained from lung transplant recipients who underwent lung transplantation at UCSF and UCLA from January 2, 2016 to February 20, 2020. BAL was collected on postoperative day 1 via bronchoscopy using 1–2 aliquots of 20 mL saline. Supplemental Figure 1 shows the inclusion and exclusion criteria for the BAL protein and RNA-sequencing studies. Notably, this effort did not collect BAL cells. Therefore, we identified 6 recipients with severe PGD who had BAL cells available in our biobank at 2 weeks after transplant and were transplanted after January, 2021. We identified 6 recipients without PGD at this same time point who were matched 1:1 based on age, sex, transplant indication, and ethnicity. Unfortunately, 2 of the PGD samples were not included because of poor viability after thawing.

### Clinical data, outcomes, protocols.

Clinical data were available as part of the United Network for Organ Sharing database (https://unos.org/data/) or were abstracted from medical records. PGD was graded on postoperative days 0 through 3 according to international criteria (10). Severe unresolving PGD was defined as grade 2 or 3 disease on postoperative days 1, 2, and 3 (63). Otherwise, recipients were classified as having “no PGD.” Standard induction regimens and maintenance immunosuppression have been detailed previously (28, 64, 65).

### Human BAL protein quantification, RNA sequencing, and immunophenotyping.

Human BAL chemokine proteins were measured with a multiplex Luminex-based assay (Eve Technologies). RNA-sequencing data were obtained and analyzed as previously described (28). NK cells from cryopreserved BAL samples were phenotyped as described previously (64, 66). Supplemental Table 6 shows antibodies employed for BAL NK cell immunophenotyping and Supplemental Figure 9 shows the gating strategy.

### Statistics.

Data resulting from mouse experiments were analyzed with unpaired, 2-tailed Mann-Whitney *U* tests after Shapiro-Wilk test for normality. Comparisons of means in experiments with multiple groups were made with Kruskal-Wallis tests, and post hoc differences were assessed with 2-tailed Mann-Whitney *U* tests adjusted for multiple comparisons with the Benjamini-Hochberg approach. Differences between NK cell surface markers were determined with paired, 2-tailed Student’s *t* tests. Correlations between NK cell and chemokine genes were assessed with Pearson’s correlation coefficient. Results were visualized using box-and-whisker plots showing individual data points bound by boxes at 25th and 75th percentiles and medians depicted with bisecting lines.

For analyses of human BAL data, differences among cohort participant characteristics were compared using 2-tailed Student’s *t* and χ^2^ tests for continuous and categorical variables, respectively. We used multivariable logistic regression to assess the association between continuous measures and PGD adjusted for the covariates of recipient age, sex, reported ethnicity, diagnosis group, and transplant type. We used Cox’s proportional hazards models to test the association between RANTES concentration and duration of mechanical ventilation. Proportional hazards were assessed visually and with the Schoenfeld test. We visualized these data with Kaplan-Meier methods plotted with the log-rank test result. For all analyses, a *P* value of less than 0.05 was considered significant. Statistical analyses and visualization were performed in R (R Foundation for Statistical Computing, Vienna, Austria) using packages “mgcv,” “ordinal,” “ggplot,” “stringr,” “multcomp,” “survminer,” “table1,” and “ggpubr.”

### Study approval.

The UCSF and UCLA institutional review boards approved the human subject components of this study under protocols 13–10738 and 13-00462, respectively. Written informed consent was obtained from all participants prior to inclusion in the study. All animal procedures and experiments were conducted according to protocols approved by the UCSF Institutional Animal Care Use Committee.

### Data availability.

Human genomics data are available in the European Molecular Biology Laboratory Bioinformatic Institute’s ArrayExpress database (https://www.ebi.ac.uk) under accession no. E-MTAB-12282. All supporting data are available in the Supporting Data Values file, main text, the supplemental materials, or upon request from the corresponding author.

## Author contributions

Order of authorship was determined based on relative contribution to the overall project. DRC conceptualized the study. DRC, JRG, SJC, JS, and LLL developed the methodology. JS, DRC, PW, AS, FL, OAA, YG, SJC, SRH, JAG, LLL, MEK, NAK, RS, AV, JK, SSW, and JAB carried out the investigation. DRC, JS, YG, and JPS formally analyzed the data. DRC and JS generated figures. DRC, JRG, MRL, and JPS acquired funding. DRC and JS wrote the original draft of the manuscript, which was reviewed and edited by DRC, JS, JRG, JPS, JB, SSW, and LLL.

## Supplementary Material

Supplemental data

Supporting data values

## Figures and Tables

**Table 1:**
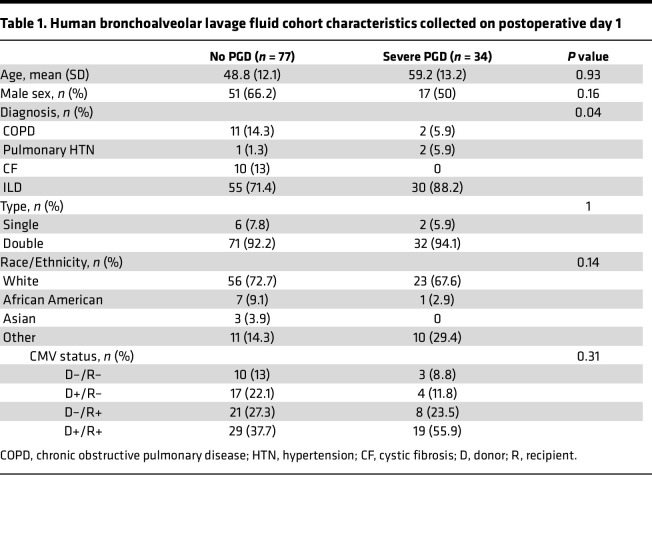
Human bronchoalveolar lavage fluid cohort characteristics collected on postoperative day 1

## Version 1 10/03/2023

In-Press Preview

## Version 2 11/08/2023

Electronic publication
