# Supplementary material for: CCR5 drives NK cell–associated airway damage in pulmonary ischemia-reperfusion injury
Source: JCI Insight. 2023 Nov 8;8(21):e173716. doi: 10.1172/jci.insight.173716 (PMC10721259; doi:10.1172/jci.insight.173716)
Supplement: Supplemental data [file jciinsight-8-173716-s056.pdf]

SUPPLEMENTAL DATA

Supplemental Figure 1. CONSORT diagram

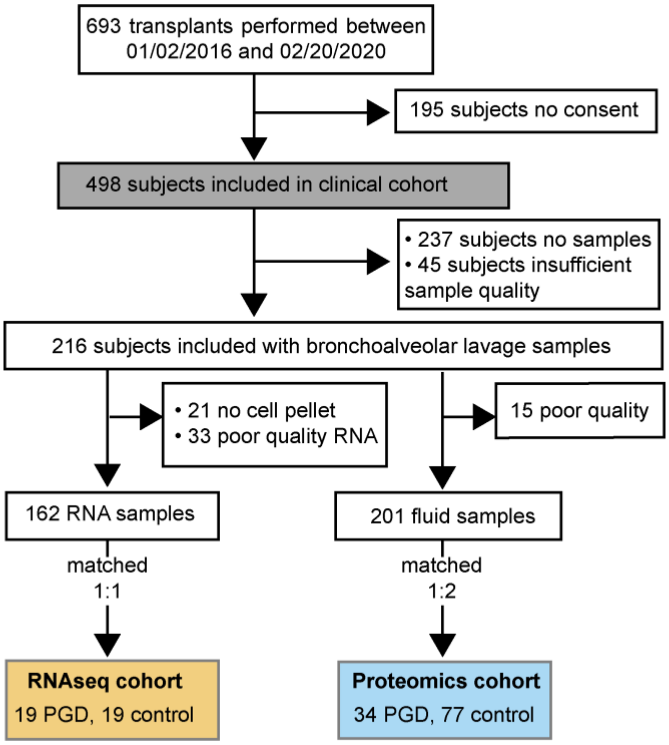

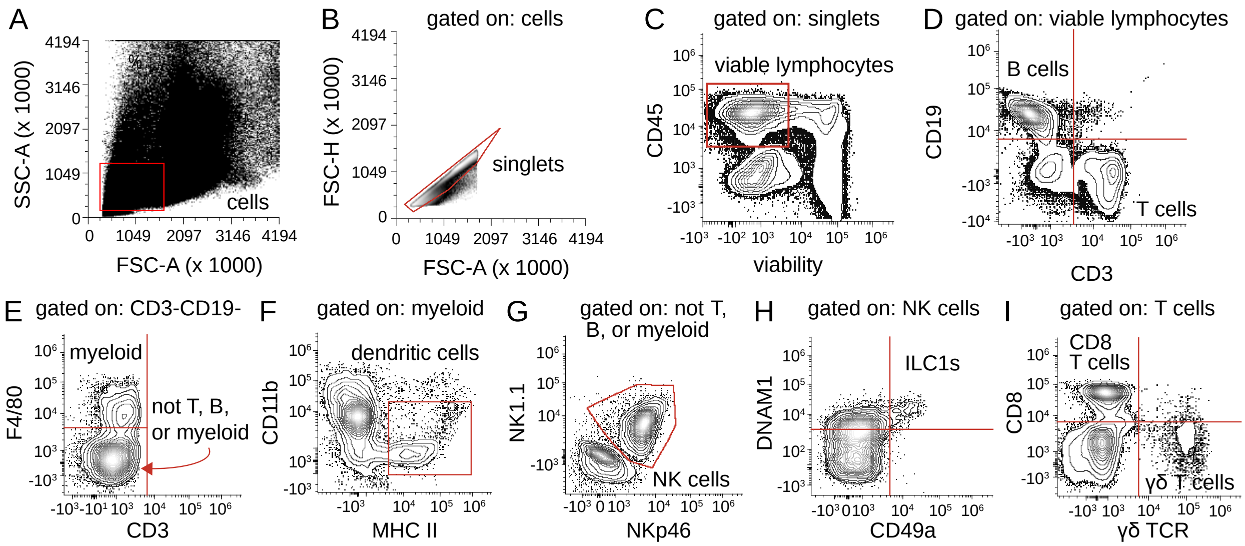

Supplemental Figure 2. Spectral Flow cytometry gating strategy. (**A**) cells were identified by side scatter (SSC-A) and forward scatter (FSC-A). (**B**) Doublets were excluded. (**C**) Viable lymphocytes were identified by exclusion of viability dye and presence of CD45 (**D**) CD19+CD45+ cells were identified as B cells and T cells were identified as CD45+CD19-CD3+. (**E**) Myeloid cells (CD3-CD19-CD45+F4/80+) cells and non-myeloid non-B cell and non-T cells were identified (CD3-CD19-CD45+F4/80-). (**F**) macrophage cell gating. (**G**) NK cells were defined as lineage negative and NKp46+NK1.1+. (**H**) ILC1s were defined as CD49a+DNAM1+ and (**I**) T cells were further subdivided based on CD8 and $\gamma\delta$ TCR.

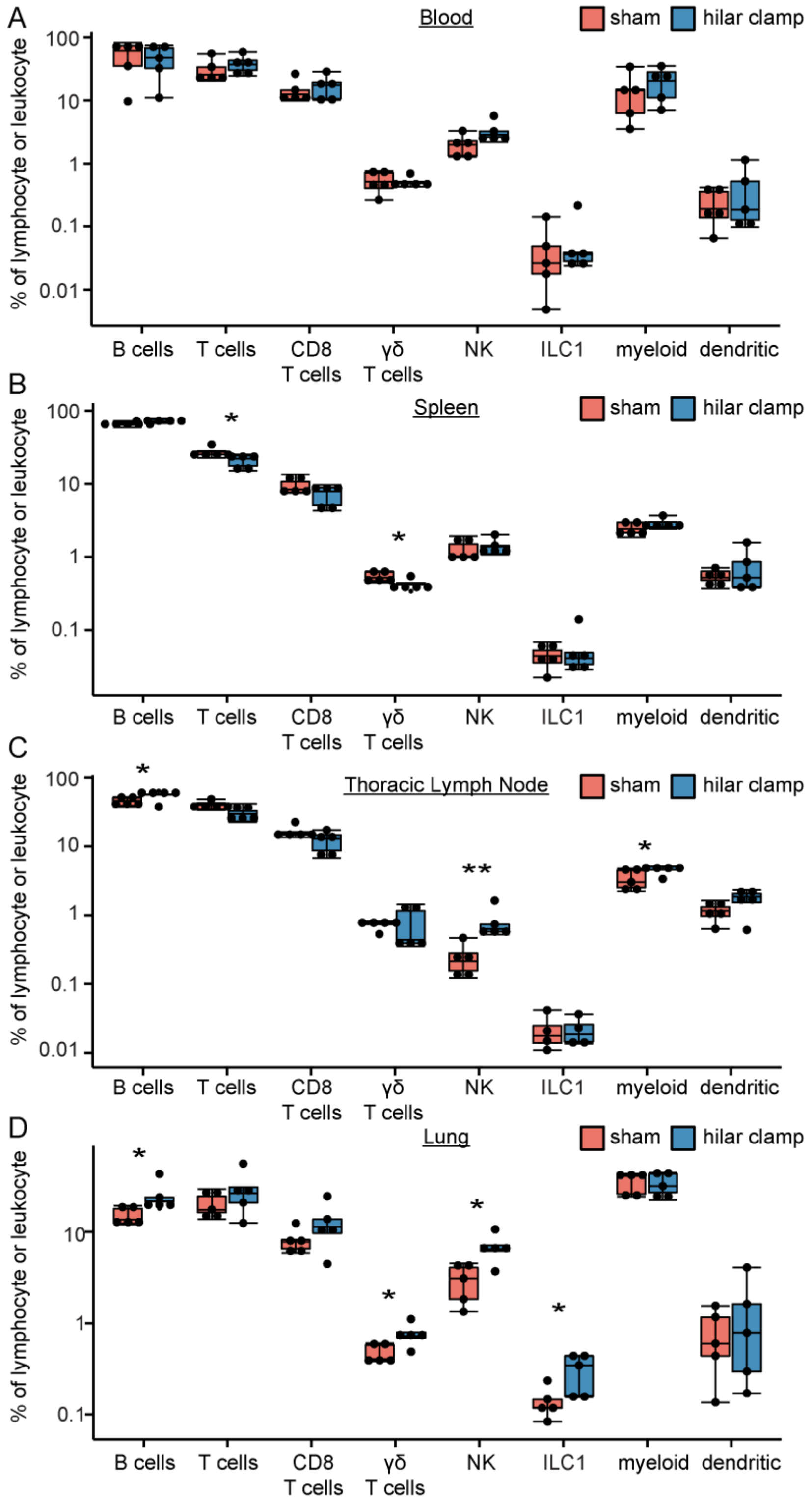

Supplemental Figure 3. Mouse cell frequencies during IRI by tissue type. We performed HC (n = 5) and sham (n = 5) procedures and collected samples at 4 hours after hilar suture release. We quantified B cells (CD45+CD19+), T cells (CD3+CD45+), CD8+ T cells (CD3+CD45+CD8),

γδ T cells (CD3+CD45+CD8+ γδ TCR+), NK cells (CD45+/CD19-/F480-/CD3-CD45+NKp46+NK1.1+), ILC1s, myeloid cells (lineage-F4/80+), and dendritic cells (lineage-F4/80+MHCII+) via spectral flow cytometry across blood, spleen, thoracic lymph node (LN) and lung tissues. Displayed are (**A**) Blood, (**B**) Spleen, (**C**) Thoracic lymph node, and (**D**) Lung tissue). Summary data are displayed with box and whisker plots illustrating individual data points, bound by boxes at 25th and 75th percentiles, and with medians depicted with bisecting lines. Differences were assessed using the Mann-Whitney U test with Benjamini-Hochberg corrections for multiple comparisons. P values; * < 0.05, ** < 0.01.

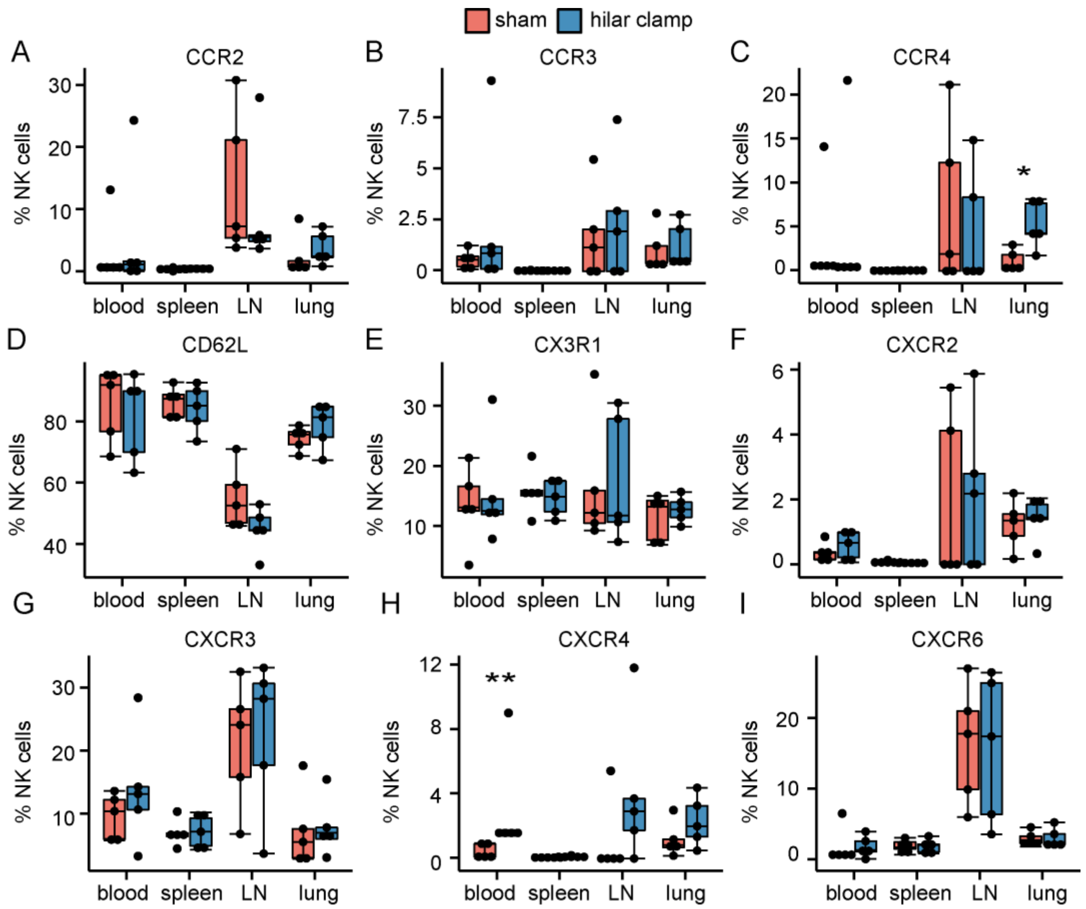

Supplemental Figure 4. Mouse NK cell chemokine receptors by tissue type during IRI or sham. We performed HC (n = 5) and sham (n = 5) procedures and quantified chemokine receptors on NK cells with spectral flow cytometry in blood, spleen, thoracic lymph node (LN), and lung tissues collected 4 hours after hilar suture removal. Chemokine receptors and trafficking molecules are shown: (**A**) CCR2, (**B**) CCR3, (**C**) CCR4, (**D**) CD62L, (**E**) CX3R1, (**F**) CXCR2, (**G**) CXCR3, (**H**) CXCR4, and (**I**) CXCR6. Summary data are displayed with box and whisker plots illustrating individual data points, bound by boxes at 25th and 75th percentiles, and with medians depicted with bisecting lines. Differences were assessed using the Mann-Whitney U test with Benjamini-Hochberg corrections for multiple comparisons. P values; * < 0.05, ** < 0.01.

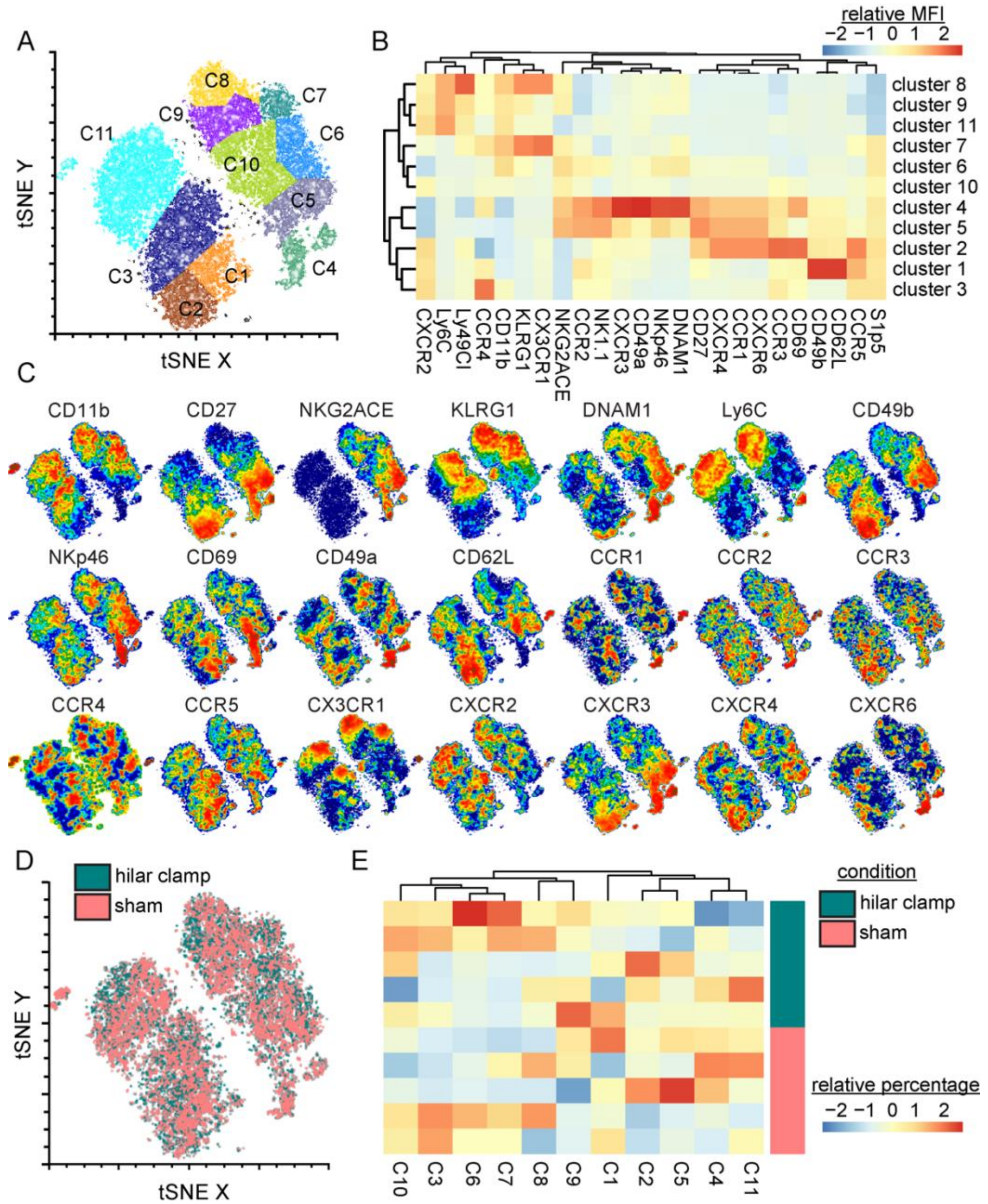

Supplemental Figure 5. Mouse NK cell chemokine receptor phenotypes. We performed HC (n = 5) and sham (n = 5) procedures and quantified immune cell phenotypes with spectral flow cytometry collected from lungs 4 hours after hilar suture removal. (**A**) tSNE plot of clusters from NK cells in the lung generated via unsupervised dimensionality reduction. (**B**) Heatmap showing MFIs of surface markers by cluster assignment. (**C**) tSNE plots of surface markers. (D) tSNE plot

shaded by hilar clamp or sham condition in the lung and (**E**) heatmap of clusters by condition in the lung.

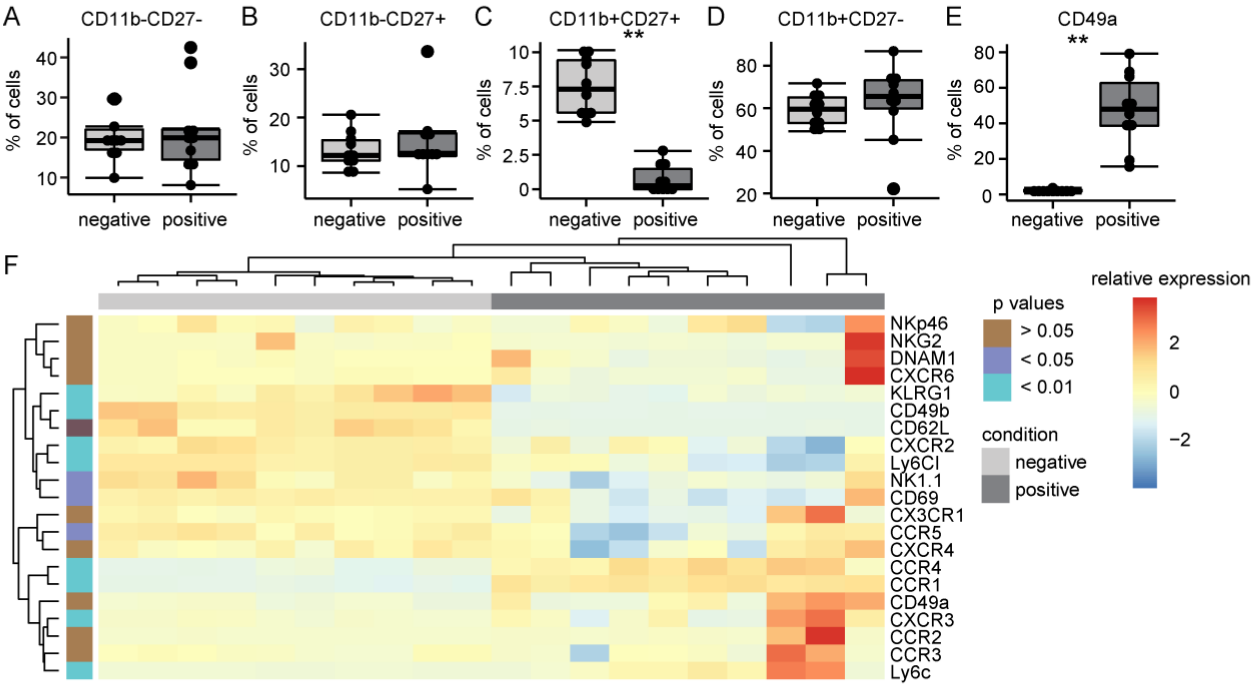

Supplemental Figure 6. Mouse NK cell CCR1 phenotypes. We performed HC (n = 5) and sham (n = 5) procedures and quantified NK cells and their phenotypes via spectral flow cytometry across blood, spleen, thoracic lymph node (LN) and lung tissues collected 4 hours after hilar suture removal. **(A-D)** We quantified maturation states of CCR1+ and CCR1- NK cells. (**E**) Frequencies of CD49a on CCR1+ and CCR5- NK cells. (**F**) Heatmap of MFI's of additional markers of NK cell activation. Summary data are displayed with box and whisker plots illustrating individual data points, bound by boxes at 25th and 75th percentiles, and with medians depicted with bisecting lines. Differences were assessed using the Mann-Whitney U test with Benjamini-Hochberg corrections for multiple comparisons. P values; * < 0.05, ** < 0.01.

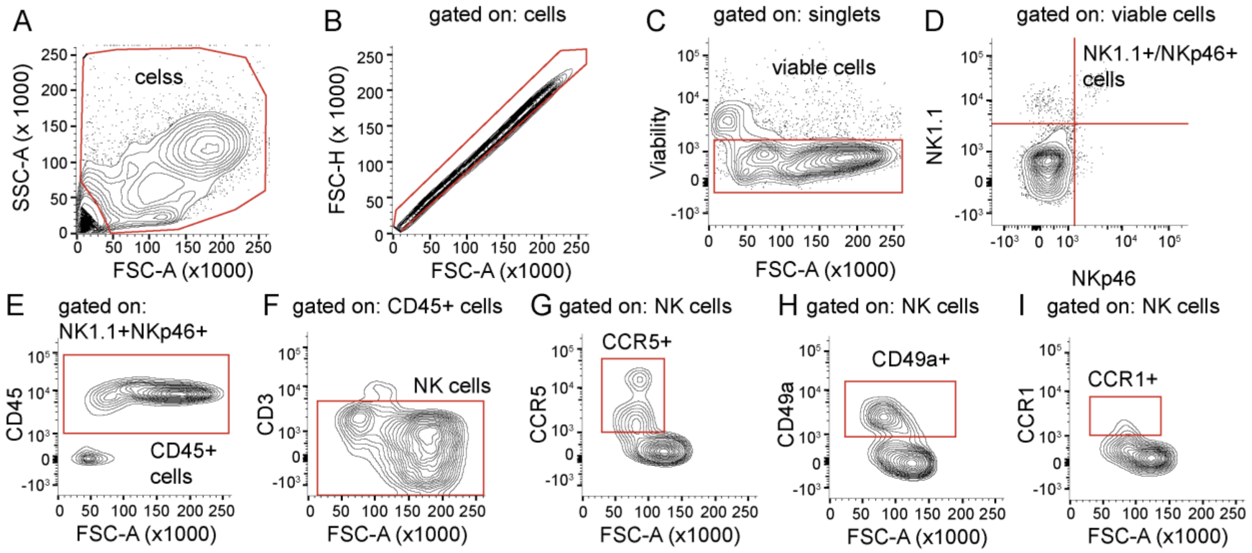

Supplemental Figure 7. Gating strategy for CCR5 immunophenotyping. (**A**) Cells were identified by side scatter (SSC-A) and forward scatter (FSC-A). (**B**) Doublets were excluded. (**C**) Viable cells were identified by exclusion of viability dye. (**D**) Cells were selected for NK cell markers (**E**) Viable NK1.1+NKp46+ cells were selected for CD45 expression, (**F**) NK cells were identified as viable NK1.1+NKp46+CD45+CD3- cells. NK cell subsets were identified by (**G**) CCR5, (**H**) CD49a, and (**I**) CCR1.

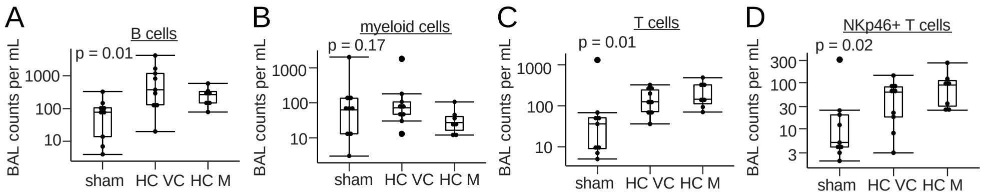

Supplemental Figure 8. Other cells in mouse BAL versus lung tissue during CCR5 blockade. C57BL6 mice were administered Maraviroc or vehicle control 24 hours and 1 hour before left lung HC and reperfusion (HC M, n = 7), vehicle (HC V, n = 8) and compared to sham procedures. Samples were collected 4 hours after left hilar suture removal. The primary outcome of total concentration of cells in the BAL were assessed for (**A**) B cells, (**B**) myeloid cells, (**C**) T cells, and (**D**) NKp46+ T cells. Summary data are displayed with box and whisker plots illustrating individual data points, bound by boxes at 25th and 75th percentiles, and with medians depicted with bisecting lines. Differences were assessed with the Kruskal Wallis test with individual p values shown in the plots.

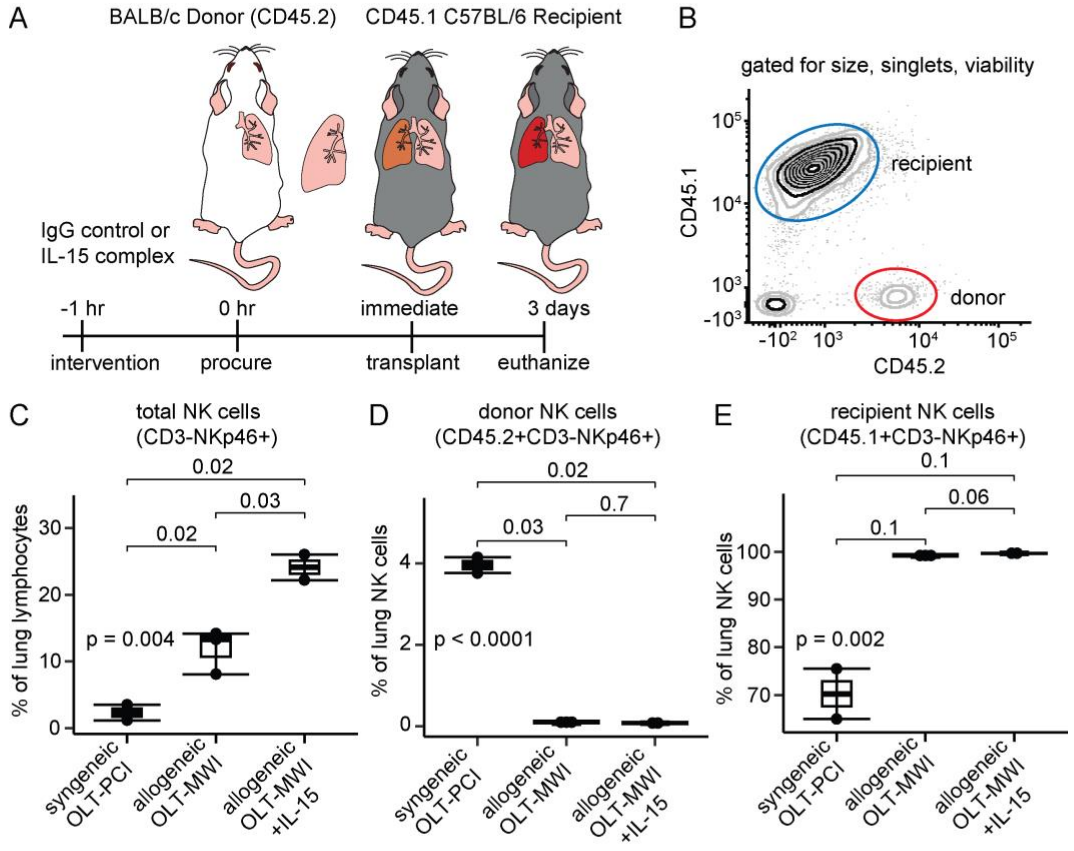

Supplemental Figure 9. Mouse NK cells in allografts versus isografts. (A) We performed allogeneic (BALB/c donor) orthotopic transplant into CD45.1 C57BL/6 recipient mice that had been pre-treated with isotype IgG (n = 3) or NK cell stimulation (IL-15 receptor complex) 1 hour before the procedure. These data were compared to previously-reported values from syngeneic OLTs (CD45.2 C57BL/6 donor transplanted into CD45.1 C57BL/6 recipient). (**B**) We identified lymphocyte origin by CD45 allotype. NK cells were assessed (**C**) as a percent of total lymphocytes with partitioning of the (**D**) donor and (**E**) recipient fractions. Summary data are displayed with box and whisker plots illustrating individual data points, bound by boxes at 25th and 75th percentiles, and with medians depicted with bisecting lines. Differences were assessed using the ANOVA test with Student's t-test for post-hoc comparisons.

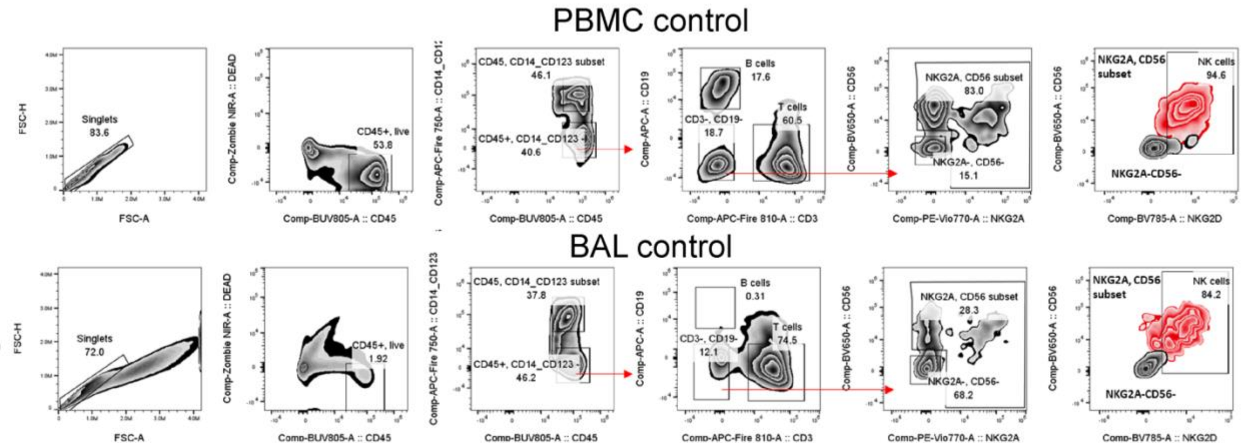

Supplemental Figure 10. Human BAL gating strategy

Supplemental Table 1. Mouse OLT-PCI top 50 differentially expressed genes

Gene	Negative log FDR-adjusted P-value	Log fold change
Crls1	10	-3.59
Fam163b	7.21	-3.32
Fgf14	8.60	-2.95
Rtn2	7.21	-2.15
Peg3os	10	-2.03
Alpl	10	-1.84
Slc39a8	7.21	-1.84
Col3a1	10	-1.76
Podn	5.90	-1.66
Igfbp4	10	-1.66
Eln	6.61	-1.61
Gas6	10	-1.56
Scara5	3.24	-1.50
Igfbp5	10	-1.49
Igfbp6	7.41	-1.45
Col1a1	9.77	-1.43
Hjurp	5.30	-1.44
Haspin	4.22	-1.43
Adamts15	4.32	-1.43
Fam205c	10	-1.39
Gm2192	7.63	-1.39
C1qa	3.61	-1.395
Svep1	4.90	-1.35
Cxcl3	6.84	1.91
Mtss1l	6.01	1.91
Igtp	10	1.91
Bcr	8.69	1.93
Ifit1	6.19	1.93
Ptprj	7.63	1.96
Cd274	10	1.97
Olfr1396	3.07	2.03
Zfp872	3.11	2.05
Tm4sf1	4.85	2.05
Rtp4	6.01	2.06
Emp1	10	2.06
Mx2	10	2.10
Ifit3	10	2.11
Gbp5	10	2.14
Znfx1	5.30	2.22
Cxcl10	8.52	2.31
Ifit2	9.60	2.35
Tnf	10	2.36
Dll1	5.85	2.39
Il1a	10	2.42
Hbegf	10	2.47
Ptges	6.32	2.57
Gbp4	10	2.71
Trappc8	5.27	3.17
Gm14023	10	3.39

Supplemental Table 2. Mouse gene scores

Gene Score	Transcripts included
NK cells	*Ccrl2, Prf1, Hbb-bs, Tgfb1, Qrfp, Ctla2a, Cd69, Il2rg, Klrk1, Prr7, Spry2, Ifngr1, Cxcr4, Fxyd5, S1pr1, Clcn3, Tm2d2, Pear1, B4galt1, Emb, Jun, Fos, Fosb, Klf2, Klf6, Jund, Junb, Nr4a1,Tsc22d3, Smad7, Stat3, Cebpb, Hes1, Hhex, Gzma, Gzmb, Zeb2, Ly6c2,Socs3, Ltb, Ly6e, P2ry10*
Chemokines	*Cxcl1,Cxcl2, Cxcl3,Cxcl5,Cxcl9, Cxcl10,Ccl2,Ccl3, Ccl4, Ccl5, Cxcl16*

Supplemental Table 3. Human bronchoalveolar lavage cell cohort characteristics

	No PGD (n = 6)	Severe PGD (n = 4)	P-value
Age, mean (SD)	60.2 (8.7)	57 (13.2)	0.71
Male Sex, N (%)	3 (50)	3 (75)	0.9
Diagnosis, N (%)			1
Pulmonary HTN	1 (16.7)	1 (25)	
ILD	5 (83.3)	3 (75)	
Type, N (%)			1
Double	6 (100)	4 (100)	
Race/Ethnicity, N (%)			
Caucasian	2 (33.3)	2 (50)	0.81
Hispanic	1 (16.7)	0 (0)	
Asian	2 (33.3)	1 (25)	
Other	1 (16.7)	1 (25)	

Supplemental Table 4. Mouse Cytek antibodies

Fluorophore	Marker	Clone	Supplier	Catalog #
BUV395	CD27	LG 3A10	BD Biosciences	740247
Viability UV	Live/Dead	N/A	Invitrogen	L34962
BUV496	CXCR2	V48-2310	BD Biosciences	750142
BUV563	CXCR3	CXCR3-173	BD Biosciences	741438
BUV615	CCR2	475301	BD Biosciences	751074
BUV737	CCR3	83103	BD Biosciences	741699
BUV805	NKG2ACE	20D5	BD Biosciences	741990
BV421	NKp46	29A1.4	BioLegend	137612
SB436	NK1.1	PK136	ThermoFisher	62-5941-82
Pacific Blue	CD69	H1.2F3	BioLegend	104524
BV510	CD19	6D5	BioLegend	115545
Pacific Orange	CD8a	5H10	ThermoFisher	MCD0830
BV570	Ly6C	HK1.4	BioLegend	128030
BV605	Ly49C/I	5E6	BD Biosciences	744029
BV650	CD11b	M1/70	BioLegend	101239
BV711	DNAM1	TX42.1	BioLegend	133609
BV785	KLRG1	2F1/KLRG1	BioLegend	138429
BB515	CCR5	C34-3448	BD Biosciences	565093
Spark Blue 550	CD3e	17A2	BioLegend	100259
PerCP	CD45	30-F11	BioLegend	103130
BB700	CD49a	Ha31/8	BioLegend	742164
PerCP/Cy5.5	CCR4	2G12	BioLegend	131220
PE	S1P5	1196A	R&D	FAB9084P
PE/Dazzle594	CX3CR1	SA011F11	BioLegend	149014
AlexaFluor594	MHC-II	M5/114.15.2	BioLegend	107650
PE/Cy5	gdTCR	GL3	ThermoFisher	15-5711-81
PE/Cy5.5	CXCR4	2B11	ThermoFisher	35-9991-80
PE/Cy7	CD49b	DX5	BioLegend	108921
APC	CCR1	S15040E	BioLegend	152504
AlexaFluor647	CXCR6	SA051D1	BioLegend	151115
APC-R700	F4/80	T45-2342	BD Biosciences	565787
APC/eF780	CD62L	MEL-14	ThermoFisher	47-0621-80

Supplemental Table 5. Mouse BAL and tissue phenotyping antibodies

Fluorophore	Marker	Clone	Supplier	Catalog #
Alexa Fluor 700	CD45	30-F11	BD Bioscience	560510
PerCP	CD3ε	145-2C11	BD Bioscience	553067
PE	NK1.1	PK136	BioLegend	108708
FITC	NKp46	29A1.4	Invitrogen	16335181
APC	CD49a	HMα1	BioLegend	142606
APC/Fire 750	CCR1	S15040E	BioLegend	152512
PE/Cyanine7	CCR5	HM-CCR5	BioLegend	107018
Brilliant Violet 605	CD19	SJ25C1	BD Bioscience	562653
Brilliant Violet 421	F4/80	T45-2342	BD Bioscience	565411
Fixable Blue Dead Cell Stain	Live\Dead		Invitrogen	L23105

Supplemental Table 6. Human Cytek antibodies

Fluorophore	Marker	Clone	Supplier	Catalog #
BUV395	CD57	NK-1	BD Bioscience	567621
Alexa Fluor 350	CD49a	1090A	R&D	FAB56761U-100UG
BV510	CCR5	J418F1	BioLegend	359128
BV605	CD62L	DREG-56	BD Bioscience	562710
BV650	CD56	HCD56	BioLegend	318344
BV711	NKp46	9E2	BioLegend	331936
BV785	NKG2D	1D11	BioLegend	320830
BUV850	CD45	HI30	BD Bioscience	612891
FITC	FcεRIγ	polyclonal	Sigma-Aldrich	FCABS400F
PE	NKG2C	134591	R&D	FAB138P
Spark YG581	CD16	3G8	BioLegend	302070
PE-eFluor 610	EOMES	WD1928	Invitrogen	61-4877-42
PE-Vio 770	NKG2A	REA110	Miltenyi Biotec	130-113-567
APC	CD19	HIB19	BioLegend	302212
APC/Fire 750	CD123	S18016F	BioLegend	396716
APC/Fire 750	CD14	M5E2	BioLegend	301854
APC/Fire 810	CD3	SK7	BioLegend	344858
Zombie NIR	Live/Dead		BioLegend	423105
